# The realism of behavioral theory-based vs. non-theory-based AI agents during a simulated infant formula shortage

**DOI:** 10.3389/frai.2026.1719703

**Published:** 2026-02-09

**Authors:** Linda Desens, Brandon Walling, Rhys O’Neill, Vanessa Howard, Mary Giammarino, Denise Scannell, Anya Kemble, Taylor Wilkerson, Nyalok Nhial, Sara Beth Elson, Maureen Leahy, Scott Rosen

**Affiliations:** The MITRE Corporation, McLean, VA, United States

**Keywords:** agentic AI, AI agent, AI agent validation, autonomous agents, behavioral theory, digital twins

## Abstract

**Introduction:**

AI-driven digital twins and autonomous AI agents are increasingly used to simulate human behavior during crises. Incorporating behavioral science frameworks may improve agent realism, but this practice is still in its infancy. This research evaluates the realism of behavioral theory-based agents in a controlled experimental design.

**Methods:**

Using a simulated infant formula shortage in South Dallas County, we compare two conditions: one with theory-based agents, and another without. Participants (human raters) assessed the perceived realism of agent decisions across both conditions.

**Results:**

Results showed significantly higher realism ratings for the theory-based agents, supporting our hypothesis.

**Discussion:**

This study constitutes an early effort to assess behavioral theory in simulation frameworks and establish a repeatable method for assessing behavioral fidelity. It provides policymakers and researchers with a theory-informed approach for enhancing AI agent realism, with the goal of increasing trust in digital twin models used for decision support in high-stakes environments.

## 1 Introduction

As autonomous agent/digital twin (DT) systems become integrated into public policy and health system planning, the credibility of agent behavior is critical. This study provides evidence for policymakers and researchers regarding the realism of behavioral theory-informed autonomous AI agents in crisis situations such as food shortages.

The ability to simulate the behavior of households related to health and social systems, and to have confidence that those simulations are realistic ones, would provide an important capacity for policymakers. However, in order to trust the simulations, there is a critical need to assess the accuracy of simulated agents during a crisis. Grounding autonomous agents in behavioral theories could improve their capacity to reflect realistic human decision-making. Our research addresses existing gaps in the literature by making the following contributions:

1. Assessing the realism of autonomous agents that are grounded in behavioral theory, comparing them with agents that are not.
2. Building an autonomous agent/DT simulation related to food access.

We use this research question: “Do participants perceive theory-based AI agents as behaving more realistically than non-theory-based agents?”

This study highlights the importance of incorporating behavioral theory in the design of AI agents. AI agent modeling and simulation should advance from a rule-based decision tree to agent designs that are grounded in empirically validated behavioral theory. To achieve greater realism, agents should be designed with socially adaptive mechanisms that enable them to adjust their strategies based on evolving human norms, values and behaviors to reflect realistic human behavior (Rahwan et al., 2019) and internal processes such as cooperation that reflect human behavior (Diau, 2025). Agents should include reflective reasoning, goal representations, memory, and behavioral theoretical grounding to maintain consistency and behavioral coherency over time (Park et al., 2023). Capturing these nuances in a simulated environment is essential to ensure that autonomous AI agents realistically reflect the decision-making patterns of the populations they represent. In this study, incorporating well-established behavioral theories into agent design ensures that simulated actions are grounded in how people react during a crisis.

This study models the behavior of households through the use case of an infant formula shortage. Food security is shaped by an interplay of policy, economic, geographic, and social systems (Roggio, 2019; Sawyer et al., 2021), as was demonstrated during the 2022 infant formula crisis. Families with infants are uniquely susceptible to disruptions across these systems, a concern that has drawn the attention of policymakers (U.S. Department of Health and Human Services, 2025; United States, 2022).

## 2 Background and literature summary

### 2.1 Previous research: autonomous agents in health and social contexts

The use of autonomous agents and DTs to simulate health and social systems has been growing, including in contexts directly related to our research, such as emergency response and urban health (Jiang et al., 2022; Subramaniyan and MohanaSundaram, 2023; Park et al., 2025). Review articles emphasize that DT use in these areas is in its infancy, and they point to technical, ethical, financial, and other barriers (Katsoulakis et al., 2024; Ringeval et al., 2025). Despite these challenges, promising case studies have validated the accuracy of DT/autonomous agent simulations (Bilal et al., 2025; Jiang et al., 2022; Park et al., 2025; Von Hoene et al., 2025) or provide a conceptual model for their use in health emergencies (Rodríguez-Aguilar and Marmolejo-Saucedo, 2020). The Bilal et al. (2025) is noteworthy in that it demonstrated predictive validity of an epidemic DT/autonomous agent model, comparing its results to observed COVID-19 data: the model closely matched actual trends in cases, hospitalizations, and deaths (Bilal et al., 2025). Assessing the accuracy of simulations is imperative to their future use: as Park et al. state, “we must develop tools and methodologies so [policymakers] know when they can, and cannot, trust these simulations” (Park et al., 2025).

Some validation methods, such as Immersive Face Validation, are primarily used to judge the believability of autonomous agents that appear in human form on-screen, for example in videos or games (Bogdanovych et al., 2016; Louloudi and Klügl, 2012). Other methods focus on agent behavior rather than visual aspects. For example, the Virtual Overlay Multi-Agent System uses supervisory agents that monitor the simulation’s adherence to predefined behavioral constraints set by human experts (Niazi, 2017). The Standardized Test Suite, which involves human success/failure ratings on agent-generated continuations of real human interaction scenarios, reveals alignment with human judgments (Abramson et al., 2022).

Other research that assesses the realism of autonomous AI agents using human raters focuses on specific contexts. For example, a growing body of work assesses the accuracy of autonomous agents for clinical decision support by comparing them to the ratings of expert physicians (Hayat et al., 2025; Ringeval et al., 2025). Park et al.’s, 2023 study is similar to our research, in that it uses human raters as participants to assess agent decisions and actions (rather than visual appearance) in simulated non-clinical environments. Park et al. (2023) created 25 generative agents that stored and synthesized memories and were embedded in a sandbox city environment. The researchers “interviewed” agents, asking questions across five categories (self-knowledge, memory, plans, reactions, and reflections). Participants then compared agent interview responses created under different agent types (full, no reflection, no reflection/plan, no reflection/plan/observation, and human baseline) and rated believability across the same five question categories. Results showed that the full generative agent type (agent ability to observe, plan, and reflect) produced the most believable behavior (Park et al., 2023). Our research builds on this work, adding the element of agents programmed to act in accordance with established behavioral theories.

### 2.2 Integration of behavioral theories

As the use of autonomous agents in simulation expands into domains such as crisis response, public health, and social policy, ensuring that agent behavior reflects realistic human decision-making becomes increasingly critical. Few studies have grounded agent logic in established behavioral science theories. Moreover, most validation efforts focus on internal consistency or task performance rather than theory-driven, externally observable and verifiable behaviors (Bogdanovych et al., 2016). This study explicitly embeds constructs from the Health Belief Model (HBM), Social Cognitive Theory (SCT), and the Theory of Planned Behavior (TPB) into autonomous agent behavior. These three theories were selected because they provide well-established frameworks for understanding how people make decisions under uncertainty, perceive risks, and respond to social and environmental cues, which are especially relevant during crises such as food shortages.

A few studies have integrated behavioral theory into agent logic or analysis of agent behavior. Chen et al.’s synthesis of existing research (2025) used Social Cognitive Theory (SCT) to suggest three dimensions that shape agent behavior over time: intrinsic attributes, environmental constraints, and behavioral feedback (Chen et al., 2025). While this is a useful perspective for analyzing the emergence of decision-making and adaptation in autonomous AI agents over time, it did not test a model that integrates behavioral theory into agent logic. One study that did so also focused on SCT, using agent-based modeling to simulate SCT’s reciprocal determinism between personal factors, behavioral patterns, and environmental events in the context of women’s career occupation choices (Mozahem, 2022). The agent-based model successfully simulated theorized aspects of SCT, demonstrating how math anxiety develops in female agents even if they are performing well in math. It also revealed that a small number of female agents exercised some control over their environment to construct their desired careers, again simulating a principle of SCT (Mozahem, 2022). Our study introduces additional elements into the use of behavioral theory for agent design, incorporating several behavioral theories and using human participants as raters for validation.

**Figure 1:**
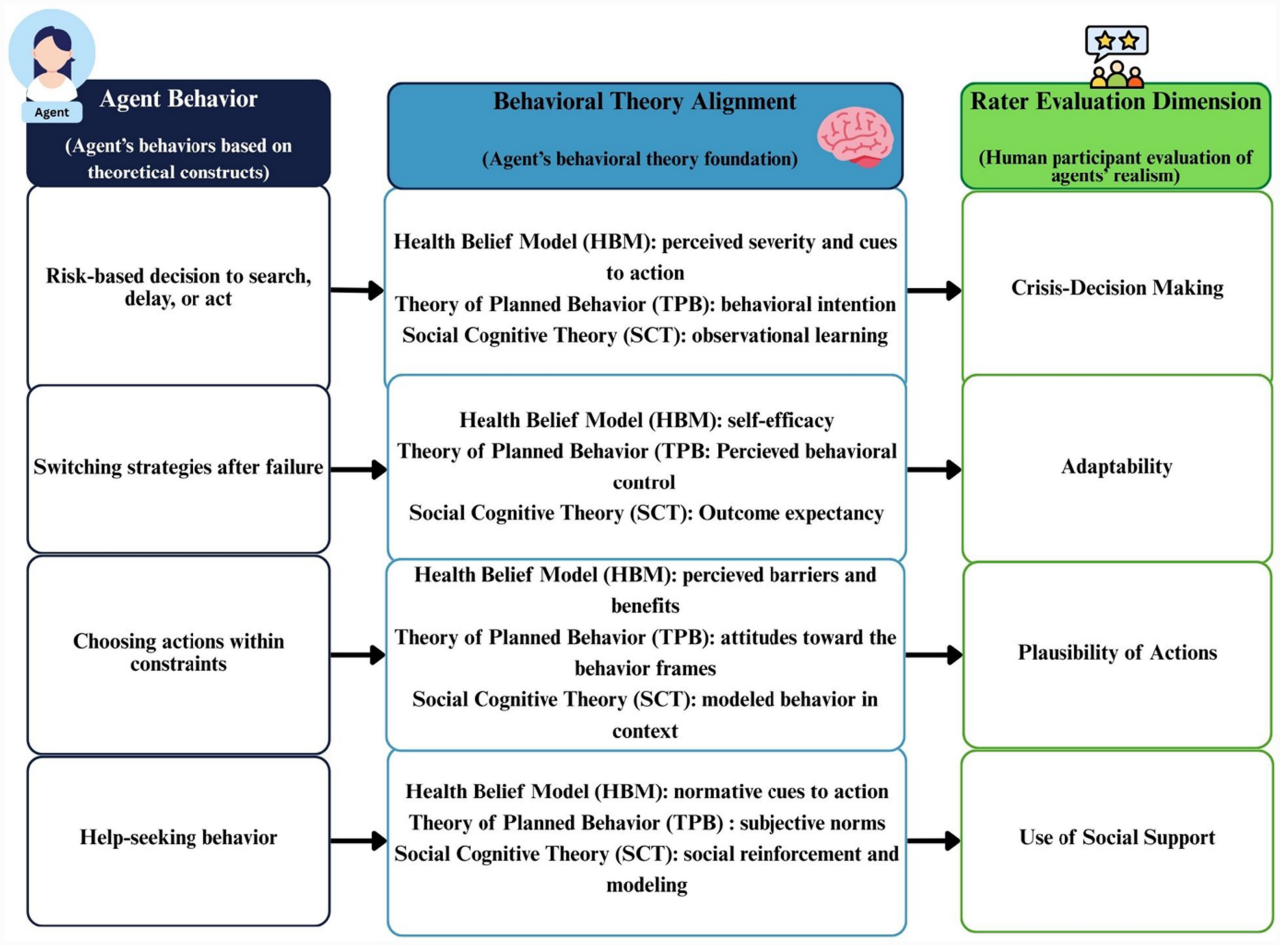
Behavioral theory alignment with agent behavior and rater evaluation.

The three behavioral theories used in this study are described in Table 1. They are operationalized in our Framework for Realistic Agent Modeling and Evaluation (FRAME) tool (Figure 1). The FRAME tool is used by participants to assess the behavioral realism of the autonomous agents. FRAME includes four evaluation dimensions (deductive foundation): (1) Crisis Decision-Making; (2) Adaptability; (3) Plausibility of Actions; (4) Use of Social Support, which are based on theoretical constructs from HBM, TPB, and SCT, as described in Table 2. These dimensions were selected because they directly map to HBM, SCT, and TPB, and are observable and assessable by participants without specialized training. The constructs listed in Table 2 align with key domains from the Health Belief Model (HBM), Social Cognitive Theory (SCT), and the Theory of Planned Behavior (TPB), as reflected in the accompanying resources column.

**Table 1:** Behavioral theories and constructs used in this research.

Behavioral theory	Theoretical constructs
Health Belief Model (HBM)	*Perceived Severity and Cues to Action*: The belief in the severity of the condition or unfavorable outcome and the associated consequences if no further action is taken. Cues to action involve the exposure to internal or external factors or stimuli that lead to action or behavior change (Alyafei and Easton-Carr, 2024; Jones et al., 2015; Orji et al., 2012). *Self-efficacy*: The belief and confidence in one’s ability to effectively perform a specific behavior (Alyafei and Easton-Carr, 2024; Jones et al., 2015; Orji et al., 2012). *Perceived barriers and benefits:* The belief in the obstacles or barriers that may prevent behavior change or health action, and the belief that the behavior change or health action could lead to positive health outcomes (Alyafei and Easton-Carr, 2024; Jones et al., 2015; Orji et al., 2012). *Normative cues to action*: The exposure to internal or external factors or stimuli that lead to action or behavior change, influenced by normative beliefs, which refer to the social norms and attitude about the specific behavior (Alyafei and Easton-Carr, 2024; Kelly et al., 2025; Orji et al., 2012).
Theory of Planned Behavior (TPB)	*Behavioral Intention:* Refers to a person’s readiness to perform a given behavior (Ajzen, 2020). *Perceived behavioral control*: The perceived control or expectancy one has over the specific behavior, often determined by control beliefs that can either promote or hinder behavior change (Bosnjak et al., 2020; Conner and Norman, 2017; Glanz et al., 2008). *Attitude toward the behavior frames*: The individual beliefs about the associated outcomes of performing a specific behavior, often influenced by behavioral beliefs specific to the consequences of a behavior (Conner and Norman, 2017; Glanz et al., 2008). *Subjective norms:* Determined by normative beliefs, which are based on whether referent others approve or disapprove of the behavior; in addition to an individual’s motivation to comply, the degree to which an individual wants to comply with referent others (Conner and Norman, 2017; Glanz et al., 2008).
Social Cognitive Theory (SCT)	*Observational Learning*: Learning to perform new behaviors through exposure, media, and watching others (modeling); influenced by attention, retention, production, and motivation (Glanz et al., 2008; Islam et al., 2023; Bandura, 2023). *Outcome expectancy*: Beliefs about the likelihood and value of the consequences of a specific behavior, often influenced by social interactions (Glanz et al., 2008; Bandura, 2023; Schunk and DiBenedetto, 2020). *Modeled behavior in context*: The demonstration of a specific behavior by a model, who is the individual being observed. Behaviors are typically imitated most when models are perceived to be like the observer (Glanz et al., 2008). *Social reinforcement and modeling*: Responses to behavior that influence how likely the specific behavior will recur; individuals are motivated to perform specific behaviors when given incentives or rewards (Glanz et al., 2008; Bandura, 2023).

**Table 2:** Evaluation dimensions and related theoretical constructs.

Evaluation dimension	Description	Construct
*Crisis Decision-making*	In real world crises, prompt and appropriate action often determines outcomes. The evaluation dimension for crisis decision-making reflects the agent’s ability to evaluate urgency and act in response to the infant formula crisis as well as their ability to change strategies after failure.	HBM: *perceived severity and cues to action;* the agent’s action is triggered by the perceived severity of the threat, their perceived susceptibility to the threat, and the cues to action such as seeing a low supply of infant formula on store shelves (Alamer, 2024). TPB: *behavioral intention*, which predicts agent action based on attitudes and norms (Bosnjak et al., 2020). SCT: *observational learning* and how it shapes decisions. (Schunk and DiBenedetto, 2020).
*Adaptability*	The adaptability dimension assesses whether agents either act or delay action predicated on risk-based decision-making.	HBM: a person’s *self-efficacy* or confidence in being able to perform a certain behavior despite barriers or obstacles (Alamer, 2024). TPB: *perceived behavioral control* construct describes the agent’s belief about their ability to carry out a certain behavior or actions (Bosnjak et al., 2020). SCT: construct emphasizes *self-regulation and outcome expectancy,* where agents adjust their behavior based on feedback and the expected results (Schunk and DiBenedetto, 2020).
*Plausibility of Actions*	The theoretical constructs for the plausibility of actions dimension checks that agents choose actions within their constraints as well as consistent with their resources and perceived outcomes.	HBM: the agent’s *perceived benefits and barriers* determine whether they pursue preventative actions (Alamer, 2024). TPB: *attitude towards the behavior* construct assesses whether a person views an action as being positive or negative (Conner and Norman, 2017). SCT: *modeled behavior in context* construct emphasizes that behavior occurs within realistic environmental constraints and reinforced outcomes (Schunk and DiBenedetto, 2020).
*Use of Social Support.*	The theoretical constructs for this dimension highlight help-seeking behavior, assessing whether agents appropriately engage with family, friends, or community networks that reflect real human coping strategies.	HBM: *cues to action through others* highlights that social cues can prompt an agent to act (Alamer, 2024). TPB: *subjective norms* emphasizes that behavior is influenced by the perceived expectations of those closest to an individual (Conner and Norman, 2017). SCT: *observational learning and social reinforcement* emphasizes that individuals learn behavior by watching others (Bandura, 2023).

The four evaluation dimensions were the foundation for the behavioral ratings used by the participants (Table 3). The tool is also based on an inductive foundation that reflects real decision contexts from the 2022 infant formula shortage (Cernioglo and Smilowitz, 2023; Keeve et al., 2024; Sylvetsky et al., 2024). For example, some of the agents continued to visit the same store to seek out formula despite past failures, indicating poor crisis decision-making or demonstrating an unrealistic strategy. Other agents sought support from social networks when they were unable to obtain formula from stores. As a final example, some agents waited until their supply of infant formula was very low before seeking additional sources, showing low adaptability.

**Table 3:** Behavioral ratings used by participants.

**Dimension**	**Description**	**Rating (1–5)**
Crisis Decision-Making	How realistic was the agent’s decision-making in response to the formula shortage?	□ 1 □ 2 □ 3 □ 4 □ 5
Adaptability	How realistic was the agent’s adjustment to changing circumstances?	□ 1 □ 2 □ 3 □ 4 □ 5
Plausibility of Actions	How realistic was the agent’s behavior compared to what a real person might do?	□ 1 □ 2 □ 3 □ 4 □ 5
Use of Social Support	How realistic was the agent’s use (or non-use) of social connections?	□ 1 □ 2 □ 3 □ 4 □ 5

Scale: 1 = Very unrealistic, 2 = Moderately unrealistic, 3 = Neither realistic nor unrealistic, 4 = Moderately realistic, 5 = Very realistic.

## 3 Methods

### 3.1 Study aims: research questions and hypothesis

The aim of this study was to empirically evaluate whether theory-based autonomous AI agents exhibit behavioral realism as perceived by participants, in contrast to autonomous AI agents without theoretical grounding. We posed the following research question and hypothesis:

- *Research Question:* Do participants perceive theory-based AI agents as behaving more realistically than non-theory-based agents?
- *Research Hypothesis:* AI agents modeled using behavioral theories will receive significantly higher realism ratings.

### 3.2 Study design

This study followed a within-subjects (repeated-measures) design where each rater (participant) evaluated the realism of autonomous agent behaviors under two experimental conditions: agents that are grounded in behavioral theory (experimental conditions) and non-theory-based agents (control condition). The independent variable was the agent type (i.e., theory-based vs. non-theory-based). The dependent variables were the realism ratings for each of the four evaluation dimensions: (1) Crisis Decision-Making; (2) Adaptability; (3) Plausibility of Actions; and (4) Use of Social Support.

#### 3.2.1 Infant formula use case and Dallas county context

Information from the 2022 formula shortage, including parental actions and the constraints on formula supply, informed our agentic AI/DT architecture. The 2022 formula shortage was precipitated by the closure of a manufacturing facility responsible for approximately 20% of the U.S. formula supply and was compounded by previous supply chain problems related to the COVID-19 pandemic (Ahmed, 2022; U.S. General Accounting Office, 2025). Policy constraints played a significant role in the formula shortage. Exclusive contracting requirements for state Special Supplemental Nutrition Program for Women, Infants, and Children (WIC) programs initially limited brand flexibility for enrolled families, until waivers were permitted. Regulatory hurdles had long made it difficult for new domestic manufacturers to enter the infant formula market, further reducing resilience (Yenerall et al., 2024). Economic and geographic factors, such as residing in food deserts or lacking transportation to travel to multiple locations to purchase formula, limited the options for many families (Blackman et al., 2022). Social networks, on the other hand, acted as protective factors, allowing some families to obtain formula through community groups and informal networks (Kalaitzandonakes et al., 2023; Keeve et al., 2024).

The reactions of parents and caregivers to the 2022 infant formula shortage determined the types of parental actions built into our agentic AI/DT architecture. These actions were codified into behavioral parameters by defining common responses and patterns from the 2022 shortage and mapping them onto the behavioral theory constructs (see Methods). For example, common parental responses from the 2022 shortage included travelling to multiple stores to find formula, searching for it online (Damian-Medina et al., 2024; Kalaitzandonakes et al., 2023) and obtaining it through family, social, and community networks, including food banks (Kalaitzandonakes et al., 2023; Keeve et al., 2024; Sylvetsky et al., 2024). Other 2022 parental actions reflected the extreme scarcity of formula that was also an element of our model. When they were unable to find formula, parents used unsafe feeding practices including watering down formula or replacing it with substitutes, such as cow’s milk or homemade infant formula (Cernioglo and Smilowitz, 2023; Sylvetsky et al., 2024).

Constraints and obstacles faced by parents in 2022 were also used to build the crisis simulation. For example, parental strategies varied based on household socioeconomic status (SES). Lower SES households receiving WIC were more likely to report difficulty obtaining formula than higher SES families in a nationally representative survey: 40% for WIC households compared to 35% for other households (Keeve et al., 2024). Higher SES households were more likely to have the resources to travel to find formula: over a 24-h period, 29% of these households visited four or more stores and 26% traveled more than 20 miles to purchase infant formula (Damian-Medina et al., 2024). In qualitative interviews, parents of varying SES described spending “exorbitant amounts” of time and energy trying to locate formula (Sylvetsky et al., 2024).

WIC households faced constraints that other households did not: they were unable to switch brands using WIC vouchers until the Food and Nutrition Service offered state WIC agencies temporary waivers of program rules to allow purchase of alternate sizes, forms, or brands of infant formula during the shortage (U.S. General Accounting Office, 2025). Lower SES families were more likely than higher SES families to use unsafe feeding practices (Cernioglo and Smilowitz, 2023; Damian-Medina et al., 2024). Parents of varying SES felt that their infant received fewer calories due to not having enough formula, resulting in what they perceived as inadequate weight gain (Sylvetsky et al., 2024). These real-world behaviors highlight how structural constraints, socioeconomic status, and caregiver perceptions shaped household responses during the shortage.

Dallas County, Texas, which has a higher child food insecurity rate than the nation as a whole, provides a meaningful setting for our simulation. Food insecurity is defined as “the limited or uncertain availability of nutritionally adequate and safe foods or limited or uncertain ability to acquire acceptable foods in socially acceptable ways” (U.S. Department of Agriculture, 2025b). It encompasses both insufficient food quantity and compromised dietary quality and can have lasting developmental and health consequences for children (Gallegos et al., 2021; Thomas et al., 2019). Child food insecurity in Dallas County was 24.5% in 2023 compared to the U.S. rate of 17.4%, and household food insecurity was 19.2% compared to the U.S. rate of 14.3% (Feeding America, 2025).

Dallas County has one of the highest WIC participation rates in Texas, and the county’s poverty rate was 13.8% compared to the U.S. rate of 12.5% in 2023 (U.S. Census Bureau, 2024). Multiple census tracts in the county are considered as having low income and low access to food (U.S. Department of Agriculture, 2025a) and were formerly called food deserts (U.S. Department of Agriculture, 2025b). Dallas County clearly faces the interplay of factors that were needed for our simulation.

#### 3.2.2 Participants

A convenience sample of 34 adult raters was recruited from a large non-profit organization. This sample size was selected based on *a priori* power analysis. Assuming a medium effect size (*Cohen’s d* = 0.5), alpha = 0.05, and desired power = 0.80, a minimum of 34 raters was required for a two-tailed paired-samples *t*-test (Faul et al., 2009). Although most participants had a behavioral health or public health background, no specialized training in the behavioral and social sciences or artificial intelligence was required. The rating dimensions were designed to be easily understandable by lay raters.

#### 3.2.3 Digital twin and agent construction

Agent behaviors were simulated in a digital twin of Dallas County, Texas, during a two-week infant formula shortage.

The digital twin construction needed to duplicate Dallas County as closely as possible within the study’s limitations to provide the household agents with accurate obstacles for securing infant formula during the crisis simulation. To provide the digital twin and agentic AI accuracy within the limitations of the build, real data sets were incorporated into their structures. Federal, state, and local data were collected and organized into two categories: (1) geographic and demographic characterizations of the digital twin of Dallas County; (2) personal barriers for the AI agents.

The digital twin covers five ZIP codes in southern Dallas County (75,216, 75,241, 75,212, 75,228, 75,231). These specific ZIP codes were drawn as boundaries for where the agents would pull data on household demographics and access to formula in the area. To define the geospatial boundaries, data from U.S. Census Bureau TIGER/Line shapefiles provided accurate polygons for the ZIP codes (U.S. Census Bureau, 2024). USDA Food Access Research Atlas data (U.S. Department of Agriculture, 2025a) were incorporated to classify areas by food access categories (e.g., low-income/low-access census tracts).

Retail, care facility, and food bank locations (Points of Interest) were mapped using the USDA Supplemental Nutrition Assistance Program (SNAP) Retailer Locator and Food Environment Atlas. These locations were generated in the Dallas ZIP codes based on HRSA Data Warehouse data (Health Resources and Services Administration, 2024), Feeding America (Feeding America, 2025), 211 Texas (Texas Health and Human Services Commission, 2025), and state WIC program listings (U.S. Department of Agriculture, Economic Research Service, 2025). The locations to obtain formula in each ZIP code were given appropriate operating hours, formula supply levels, and crisis-response triggers for critically low households.

A supply chain network layer was integrated into the functionality of the simulation modeling to more accurately depict the flow and disruption of infant formula from manufacturers downstream to consumers. The layer incorporated manufacturer production rates, distributor restock intervals, and interval levels. It produced store stock inventory status, restock timing, and household level access that all impacted the agent purchasing capacity. Manufacturers of infant formula, specifically Abbott and RBMJ, were based on 2022 market share data (U.S. Food and Drug Administration, 2023). The supply chain model was designed to implement additional disruptions and supply surges, such as temporary plant closures and emergency importation protocols. To model the retail inventory dynamics, shelf-space allocation rules for WIC products, price variability based on Nielsen retail scanner data, and spiked panic-buying behavior when consumer visible inventory fell below 30% of baseline levels were all incorporated into the supply chain logic. Tariff increases and FDA waivers were used as policy intervention inputs to also serve as realistic impacts to infant formula availability.

Transportation options were modeled into this layer of the digital twin to provide realistic barriers for accessing locations with infant formula supply. This layer integrated Dallas Area Rapid Transit (DART) data (Dallas Area Rapid Transit, 2025) to provide realistic geospatial relevance for the household agents, including nine bus routes, two light rail lines, and 12 major transit stops across the target ZIP codes. Routes had a daily schedule, travel frequencies, and appropriate fares.

To model temporal access barriers, caregiver employment schedules were sourced from American Community Survey (ACS) employment data (U.S. Census Bureau, 2025) and integrated into the digital twin. This feature provided timeframe limitations to obtain formula for household agents. Local retail stores and healthcare offices that held infant formula inventory in the household agent’s specific ZIP codes operated on realistic schedules and were shifted when triggered with crisis-adjusted extensions. This trigger expanded clinic hours during emergency response activation.

#### 3.2.4 Evaluation scale

Participants used the evaluation scale to assess the perceived realism of the agent’s behavior across the four dimensions by selecting a score on the 5-point Likert scale for each of the four dimensions: Crisis Decision Making, Adaptability, Plausibility of Actions, and Use of Social Support (see Table 3). Inter-rater reliability was assessed to evaluate the consistency among mean realism ratings between participants. Prior to rating, participants received written guidance on how to interpret each FRAME dimension and the 1–5 scale. A score of ‘5’ indicated that the agent’s response was highly consistent with how a real person under similar crisis conditions would be expected to behave (i.e., very realistic), whereas a score of ‘3’ reflected behavior that was neither clearly realistic nor unrealistic. For example, on the Adaptability dimension, an agent proactively changing strategy after encountering an obstacle (e.g., immediately seeking alternative locations or transportation upon finding a store out of formula) was rated a ‘5’. In contrast, waiting until formula was nearly depleted before taking action was rated lower (1–2), and reactive but delayed adaptation represented a mid-range (‘3’) score. Comparable examples were described for the remaining dimensions (Crisis Decision-Making, Plausibility of Actions, Use of Social Support) to ensure consistent application across raters.

#### 3.2.5 Procedure

Participants were recruited via an email that described the study and included the informed consent. Upon receipt of their signed informed consent, the participants were sent a link to the digital form that included vignettes and the evaluation tool (see Appendix). Vignettes provided a summary of each agent’s actions over the two-week simulation and did not include any theoretical labels.

Each participant evaluated four vignettes from each condition, for a total of eight. The conditions were: (1) theory-based agents whose behaviors consisted of theoretical constructs from HBM, TPB, and SCT; and (2) non-theory-based agents that followed simple heuristics with no theoretical grounding. Each participant evaluated an equal number of vignettes per condition, presented in a randomized order using the evaluation scale. To mitigate order effects, participants were divided into subsets with different vignette presentation orders. This full counterbalancing ensured that each vignette appeared in each position in the sequence an equal number of times across participants.

### 3.3 Agent descriptions

#### 3.3.1 Household agent descriptions

The theory-based agents in this study were designed to simulate realistic human decision processes by integrating constructs from HBM, TPB, and SCT. The agent’s decision process was defined by the Belief–Desire–Intention (BDI) framework, allowing agents to reflect on the situation, choose strategies that best fit their situation, and plan day-to-day in response to changing information (Archibald et al., 2023). HBM, TPB, and SCT constructs were mapped into the theory-based household agents via agent variables that used federal survey data for calibration. These variables were embedded into a Belief–Desire–Intention cycle: beliefs reflected updated perceptions of threat, barriers, and social norms; desires represented the goal of securing infant formula; and intentions were expressed as strategy choices. Available strategies included visiting stores, contacting WIC vendors, seeking social help, searching online, traveling further distances, turning to food banks, or waiting. Which option was chosen depended on the agent’s theory-based traits, household attributes, and the urgency of the situation. Non-theory-based control agents followed a simple rule-based decision tree without beliefs, social influence, or adaptive updates. This contrast provided a clear experimental control for evaluating whether theory-driven constructs improved perceived realism. Agents follow a structured BDI-style decision cycle where beliefs are updated from environmental and social cues, then modulated by stable trait-derived weights. These weighted beliefs produce intention scores that determine action choice. To avoid deterministic outcomes, a small bounded stochastic term is applied during intention formation, ensuring behavioral variation while preserving theoretical alignment. No reinforcement learning is used, and agent traits remain fixed for the duration of the scenario. A representative summary of the code is provided in Table 4.

**Table 4:** Agent decision cycle (representative).

AGENT_DECISION_CYCLE (agent, environment):
# 1. Perceptioncues = OBSERVE(environment)social_inputs = OBSERVE_SOCIAL_CONTEXT(agent)# 2. Belief Updatingbeliefs = UPDATE_BELIEFS(prior_beliefs = agent.beliefs,cues = cues,social_inputs = social_inputs)# 3. Trait-Based Weightingweighted_beliefs = APPLY_TRAIT_MODIFIERS(beliefs = beliefs,traits = agent.traits)
# 4. Intention Formation
intention_scores = COMPUTE_INTENTIONS(
weighted_beliefs = weighted_beliefs,
situational_cues = cues
)
# 5. Controlled Stochastic Variation
intention_scores = ADD_BOUNDED_NOISE(
intention_scores
)
# 6. Action Selection
selected_action = SELECT_HIGHEST_SCORE(
intention_scores
)
# 7. Execution
EXECUTE(agent, selected_action)
UPDATE_MEMORY(agent, cues, selected_action)
return selected_action

*Worked example: theory-based agent decision process.*

This example illustrates how a single theory-based household agent translates scenario inputs into a decision using behavioral theory constructs within the BDI framework.

During the simulation, the agent encounters repeated store visits where infant formula is unavailable, while its household supply is steadily declining. The agent also observes information broadcasts indicating prolonged supply disruption and receives informal updates from nearby households experiencing similar difficulty.

These environmental cues are interpreted as theory-aligned perceptions rather than direct action triggers. Under the Health Belief Model, the persistent lack of formula availability and declining household supply increase the agent’s perception of severity and susceptibility. Under the Theory of Planned Behavior, limited transportation options and work schedule constraints reduce perceived behavioral control for extended store searches. Under Social Cognitive Theory, exposure to other households successfully obtaining formula through community resources increases the perceived effectiveness of help-seeking behaviors.

These perceptions update the agent’s internal belief state within the BDI cycle. The agent’s desire remains focused on securing sufficient infant formula, but its intention shifts from repeated store visits toward seeking assistance through social and institutional channels. The agent then selects an action aligned with this intention, such as contacting a food bank or community organization, subject to environmental constraints such as operating hours and eligibility.

This process allows agent behavior to evolve over time in response to changing conditions while remaining grounded in established behavioral theory, producing actions that reflect realistic human decision-making under crisis conditions rather than fixed rule execution.

*A complete mapping of scenario* var*iables to behavioral theory constructs and decision effects is provided in* Table 5.

**Table 5:** Mapping of scenario inputs to behavioral theory constructs and agent decision effects.

Scenario input or cue	Health belief model (HBM)	Theory of planned behavior (TPB)	Social cognitive theory (SCT)	Resulting behavioral effect
Declining household formula supply	Increased perceived severity and susceptibility	Strengthened behavioral intention to act	Heightened outcome expectancy	Earlier escalation of search or help-seeking behavior
Repeated store stockouts	Cues to action triggering reassessment	Reduced perceived behavioral control	Observational learning from failed attempts	Shift away from repeated store visits
Limited transportation access	Increased perceived barriers	Lower perceived behavioral control	Constraint on modeled behavior in context	Preference for nearby or non-travel-based options
Information broadcasts about shortage	External cues to action	Normative influence shaping intention	Reinforcement of shared social understanding	Increased urgency and strategy change
Observing others obtain formula through support networks	Normative cues to action	Subjective norms favoring help-seeking	Observational learning and social reinforcement	Increased likelihood of seeking social or institutional support
Prior failed strategies	Reassessment of perceived benefits vs. barriers	Adjustment of attitudes toward alternatives	Feedback-driven adaptation	Abandonment of ineffective strategies

As the control, non-theory-based agents made decisions based on rule-based heuristics. The agents followed a fixed decision tree that had a baseline functionality: when inventory was critically low and transport was available the agent chose a store visit, and when transport was not available the agent would ask for assistance. The decision tree allowed different choices under different levels of urgency (moderate and low). However, the non-theory-based agents did not have choices that involved belief updates, social influence, or adaptive weighting. This created a clear control condition for evaluating the extent to which theory-driven constructs built into the agents improved perceived realism in simulated crisis scenarios. For a comparison of theory-based versus non-theory-based agents (see Table 6).

**Table 6:** Comparison of theory-based vs. non-theory-based agents.

Feature	Theory-based agents	Non-theory-based agents
Behavioral Theory Integration	Fully integrates HBM, TPB, and SCT constructs (e.g., perceived severity, self-efficacy, intention formation, subjective norms).	Operates using fixed utility maximization with invariant preference weights for formula acquisition, transportation costs, and time constraints.
Belief–Desire–Intention (BDI) Processing	Employs BDI architecture for belief updates, intention generation, and memory consolidation.	Employs deterministic rule-based decision trees with static thresholds for resource-seeking behaviors and fixed action sequences.
Output	Produces nuanced, theory-driven actions reflecting realistic human crisis decision-making.	Produces simple, deterministic actions lacking behavioral realism.

The non-theory-based agents were intentionally designed as a simple, transparent baseline rather than as a behaviorally realistic or predictive model. Their purpose was to provide a deterministic comparison condition that reflects common rule-based approaches used in agent-based modeling, allowing the study to isolate the contribution of behavioral theory to perceived realism while holding environmental conditions constant.

The decision tree logic was reviewed for face validity by domain experts with experience in public health response, food access systems, and crisis planning to ensure that the modeled actions were plausible and representative of commonly assumed baseline behaviors. No empirical calibration or benchmarking against real-world action frequencies was performed, as the study evaluates relative perceived realism between agent types rather than absolute behavioral accuracy.

Attributes for household agents were parameterized using data from the Food Acquisition and Purchase Survey (U.S. Department of Agriculture, Economic Research Service, 2025) and the ACS (U.S. Census Bureau, 2025) to assign household size, caregiver employment type, income level, poverty, and car ownership rates at the ZIP code level. These variables impact the agent’s level of need (one or multiple child home), ability to secure formula during working hours or the need to find a time window outside of regular business hours, and transportation to reach different stores beyond the primary convenient choices. Behavioral Risk Factor Surveillance System (U.S. Centers for Disease Control and Prevention, 2025) indicators (e.g., caregiver stress and nutrition-related health behaviors) were incorporated to influence agent tendencies toward risk-averse or adaptive decisions, with higher stress impacting the choices to make longer trips to visit multiple stores. Program participation data from WIC and SNAP reports (U.S. Department of Agriculture, Food and Nutrition Service, 2025) determined purchasing power, WIC voucher eligibility, and access to retailer networks which constrain purchase channels and store choices.

Social networks are implemented through geographic proximity assignment, where agents connect to service providers within ZIP code service areas and defined distance thresholds. Agent interactions are further structured by functional groupings based on institutional relationships, creating realistic service-seeking patterns, coordination networks, and hierarchical reporting structures for crisis response.

#### 3.3.2 Service and support agents

The simulation included a set of agents that represented services and support mechanisms that would be called upon during an infant formula shortage. As an extension of the environmental layers built into the digital twin, these systems agents included food banks and community organizations, healthcare providers, or government programs. Each agent was designed with pertinent data and practices so that their behavior aligned with how these systems realistically function. By including these agents, the model captures both the pressures households face and the institutional responses that impact access during a crisis.

Three agent types were created to model crisis response systems:

- The NGO foodbank agent leveraged food pantry locations from 211 Texas (Texas Health and Human Services Commission, 2025) and FEMA Community Resilience Index data (Federal Emergency Management Agency, 2025) to model organizational responsiveness to high-stress conditions. These agents distributed emergency formula products when households were critically low, avoiding starvation outputs in the simulation.
- The health system agents incorporated CDC Population Level Analysis and Community Estimates (PLACES) data (Centers for Disease Control and Prevention, 2024) for local nutrition-related health needs and HRSA Data Warehouse records (Health Resources and Services Administration, 2024) to determine healthcare facility capacity and coverage. These agents provided referrals, distributed limited formula samples, activated extended hours during crises, and offered telehealth consultations.
- The policy program agents simulated state and federal program interventions using the USDA SNAP Policy Database (U.S. Department of Agriculture, Economic Research Service, 2024) and state-level WIC waiver policies (U.S. Department of Agriculture, Food and Nutrition Service, 2023). These agents found ways to mitigate the lack of access to infant formula through built-in interventions triggered when the shortage worsened. They released emergency funding, expanded WIC brand eligibility, introduced price controls, and kept household agents updated about the shortage with regular broadcasts as the crisis developed.

### 3.4 Model data and simulation construction

The digital twin construction allowed simulated conditions to be deployed to represent the 2022 infant formula shortage in five ZIP codes in Dallas County (75,216, 75,241, 75,212, 75,228, 75,231). These five ZIP codes were chosen because they had a combination of high food insecurity, WIC participation, and transportation barriers. The model integrated geospatial, retail, supply chain, transportation, and temporal layers for accuracy of the environment within the study’s limitations.

The geospatial layout was developed using U.S. Census TIGER/Line shapefiles (U.S. Census Bureau, 2024) with ACS demographic, income, and vehicle ownership data (U.S. Census Bureau, 2025; U.S. Census Bureau, 2024), and USDA Food Access Research Atlas classifications (U.S. Department of Agriculture, Economic Research Service, 2024). Retail and institutional locations were placed throughout the ZIP codes by using SNAP (U.S. Department of Agriculture, Economic Research Service, 2024) and Food Environment Atlas data (U.S. Department of Agriculture, 2025a), HRSA (Health Resources and Services Administration, 2024), Feeding America (Feeding America, 2025), and state WIC listings (U.S. Department of Agriculture, Food and Nutrition Service, 2023). Each location was given specific attributes to appropriately project its capacity, operating hours, and crisis protocols. Supply chain modeling simulated formula flows from manufacturers to retailers using 2022 market share data, plant closures, importation rules, inventory thresholds, price variability, and policy disruptions. Transportation modeling used DART schedules, routes, and fares, with constraints from car ownership and ADA accessibility. Temporal constraints reflected caregiver work schedules and realistic operating hours, creating authentic time-based barriers to formula access.

This simulation was built using Python 3.11 + and implemented with FastAPI and WebSocket communication to support scalable framework deployment and real-time streaming of the agent decision-making process. Pydantic validation ensures structured data serialization for JSON message transmission, enabling consistent output formatting for downstream analysis and vignette generation. A custom multi-agent orchestration system coordinates the parallel execution of institutional agents alongside the household agent population within the digital twin environment.

## 4 Results

To answer the research question and hypothesis comparing participant realism scores for the theory-based compared to non-theory-based agents, a paired-samples *t*-test was used. Using the paired *t*-test, we compared mean realism rates for theory-based versus non-theory-based vignettes. For additional analysis to evaluate the realism scores by each of the four dimensions, a repeated-measures ANOVA was used to further test the difference in ratings (Agent Type – theory or non-theory × Dimension). Effect sizes were calculated as *Cohen’s d* for paired-samples *t*-tests and partial eta-squared ( $\eta_{p}^{2}$ ) for repeated-measures ANOVA.

### 4.1 Overall realism ratings by agent type

The participants rated the theory-based and non-theory-based AI agents for realism across the four dimensions (see Appendix for sample rating forms). As shown in Table 7, theory-based agents received higher mean realism scores (*M* = 3.99, *SD* = 0.58, 95% CI [3.79, 4.20]) compared to non-theory-based agents (*M* = 3.41, *SD* = 1.00, 95% CI [3.06, 3.76]). Notably, the 95% confidence intervals for these two conditions do not overlap, with the lower bound of the theory-based CI (3.79) exceeding the upper bound of the non-theory-based CI (3.76), providing evidence of a reliable difference in perceived realism between theory-based and non-theory-based agents.

**Table 7:** Mean realism ratings by condition.

Condition	Mean realism score	Std. deviation	95% CI
T1	4.15	0.73	[3.90, 4.41]
T2	3.76	0.98	[3.42, 4.11]
T3	3.88	0.80	[3.59, 4.16]
T4	4.18	0.65	[3.96, 4.41]
Total Theory-Based	3.99	0.58	[3.79, 4.20]
N1	3.27	1.19	[2.86, 3.69]
N2	3.52	1.17	[3.11, 3.93]
N3	3.54	1.09	[3.16, 3.92]
N4	3.32	1.10	[2.93, 3.70]
Total Non-Theory-Based	3.41	1.00	[3.06, 3.76]

### 4.2 Inter-rater reliability

To assess the consistency of participant ratings using the FRAME tool, intraclass correlation coefficients (ICC) were calculated. Inter-rater reliability for the average ratings across participants, vignettes, and dimensions was high (ICC = 0.92, 95% CI [0.87–0.96], *p* < 0.001), suggesting that the mean realism scores across vignettes and dimensions were stable and interpretable. When evaluated by agent type, inter-rater reliability was higher for non-theory-based vignette ratings (ICC = 0.96, 95% CI [0.93–0.98], *p* < 0.001) than for theory-based vignettes (ICC = 0.86, 95% CI [0.77–0.92], *p* < 0.001). The results also indicated high consistency among raters across dimensions (ICC = 0.94, 95% CI [0.90–0.97], *p* < 0.001).

Lower ICC values for theory-based vignettes likely reflect the greater behavioral nuance introduced by integrating constructs from behavioral theory. Some theory-driven agent behaviors were interpreted differently among raters, resulting in wider variability. In contrast, non-theory-based agents tended to follow simpler decision rules, increasing scoring consistency across raters. ICCs for all vignettes are presented in Table 8.

**Table 8:** Intraclass correlation coefficients by vignette.

Vignette	ICC	95% CI
T1	0.68	[0.46–0.82]**
T2	0.79	[0.65-0.89]**
T3	0.65	[0.41-0.81]**
T4	0.40	[−0.01-0.67]
N1	0.87	[0.78–0.93]**
N2	0.88	[0.80-0.93]**
N3	0.89	[0.81-0.94]**
N4	0.87	[0.78-0.93]**

**p < 0.001.

### 4.3 Descriptive statistics

**Figure 2:**
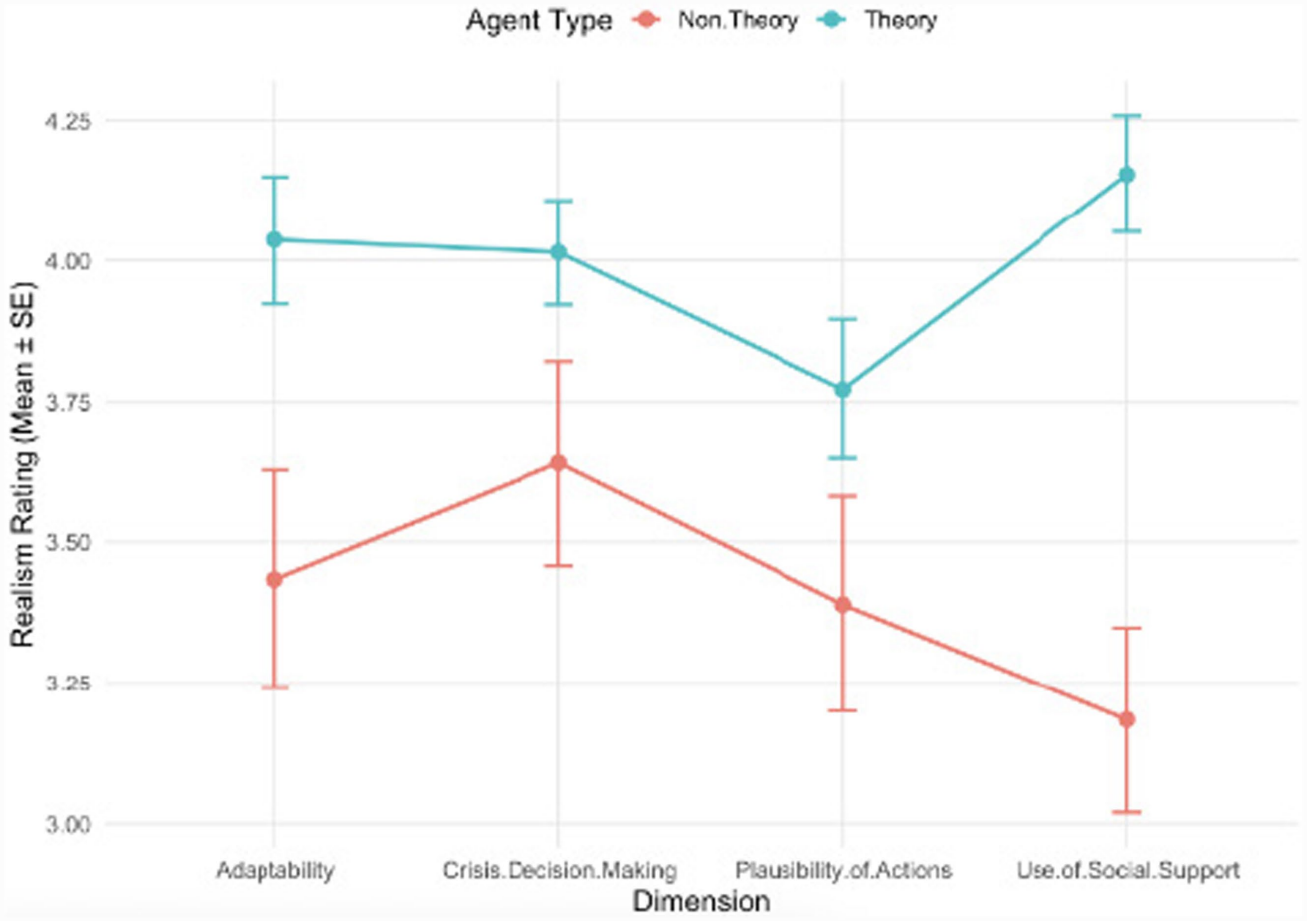
Descriptive plots of mean realism scores (theory-based vs. non-theory-based).

Tables 7, 9 present the mean realism scores, standard deviations, and 95% confidence intervals for theory-based and non-theory-based agents across all four dimensions. See Figure 2 for descriptive plots (mean + Standard Error) of the mean realism scores by dimension for theory-based versus non-theory-based vignettes. Theory-based agents consistently received higher mean scores and lower variability compared to non-theory-based agents, indicating greater perceived realism and consistency in agent behavior.

**Table 9:** Mean realism scores by dimension for theory-based and non-theory based agents.

Agents	Dimension	Mean realism	Std. deviation	95% CI
Theory-Based	Crisis Decision-Making	4.01	0.86	[3.87, 4.16]
	Adaptability	4.04	0.91	[3.88, 4.19]
	Plausibility of Actions	3.78	1.10	[3.59, 3.96]
	Use of Social Support	4.15	0.90	[4.00, 4.31]
**Total**	All Dimensions	3.99	0.96	[3.91, 4.08]
Non-Theory-Based	Crisis Decision-Making	3.64	1.23	[3.43, 3.85]
	Adaptability	3.43	1.27	[3.22, 3.65]
	Plausibility of Actions	3.39	1.32	[3.17, 3.61]
	Use of Social Support	3.18	1.20	[2.98, 3.39]
**Total**	All Dimensions	3.41	1.26	[3.30, 3.52]

### 4.4 Paired-samples *t*-tests

To test the research question and hypothesis and determine whether participants perceived the theory-based agents to be more realistic when compared to non-theory-based agents, a paired-samples *t*-test was used to compare the mean realism scores across conditions. The results indicated that the difference in realism scores across conditions (Theory-based: “T1” –” T4”; Non-Theory-Based: “N1” – “N4”) (*Mean difference* = 0.58) was statistically significant (*t* (33) = 3.13, *p* < 0.01, *Cohen’s d* = 0.54, 95%CI [0.20, 0.96]).

### 4.5 Repeated-measures ANOVA

A repeated-measures ANOVA was conducted with Agent Type and Dimension as within-subject factors. Assumptions of repeated-measures ANOVA were examined prior to interpreting the results. Mauchly’s test indicated that the assumption of sphericity was violated for Dimension (*W* = 0.61, *p* = 0.007) and for the Agent Type x Dimension interaction (*W* = 0.68, *p* = 0.029). Greenhouse–Geisser corrections were therefore applied, however, in all cases the corrected tests led to the same conclusions as the uncorrected analyses.

The ANOVA revealed a significant main effect for Agent Type [*F*(1, 33) = 9.81, *p* = 0.0036], a significant main effect for Dimension [*F*(3, 99) = 4.61, *p* = 0.0046], and a significant Agent Type x Dimension interaction [*F*(3, 99) = 11.84, *p* < 0.001]. Full ANOVA results are presented in Table 10.

**Table 10:** ANOVA results.

Effect	df	F	*p*	*η*^2^p
Agent Type	1, 33	9.81	0.0036	0.23
Dimension	3, 99	4.61	0.0046	0.12
Agent Type x Dimension	3, 99	11.84	0.000001	0.26

### 4.6 *Post-hoc* analyses

To clarify the source of the significant main effect of Dimension, pairwise comparisons using Holm-adjusted *p*-values were conducted. Averaged over Agent Type, the Crisis Decision-Making dimension differed significantly from the Plausibility of Actions dimension (estimate = 0.246, *SE* = 0.063, *p* = 0.003), while other comparisons did not reach statistical significance.

To explore the Agent Type x Dimension interaction, *Post-hoc* tests were conducted within each dimension. Participants rated the theory-based agents as more realistic than non-theory-based agents for the Adaptability dimension (estimate = 0.603, *SE* = 0.2, *p* = 0.0039) and the Use of Social Support dimension (estimate = 0.971, *SE* = 0.179, *p* < 0.001). Differences in the Crisis Decision-Making (*p* = 0.068) and Plausibility of Actions (*p* = 0.087) dimensions were not statistically significant. See Table 11 for a full report of the *Post-hoc* comparisons of agent type differences within each dimension.

**Table 11:** *Post-hoc* comparisons – simple effect of agent type within each dimension.

Dimension	Estimate	SE	*t(33)*	*P (Holm)*
Adaptability	0.603	0.20	3.01	0.0049
Crisis Decision-Making	0.375	0.20	1.89	0.0679
Plausibility of Actions	0.382	0.22	1.77	0.0865
Use of Social Support	0.971	0.18	5.43	< 0.0001

Complementary comparisons within each Agent Type showed that, for Theory-Based agents, the Plausibility of Actions dimension differed significantly from the Crisis Decision-Making dimension (estimate = 0.243, *SE* = 0.074, *p* = 0.010) and from the Use of Social Support dimension (estimate = 0.382, *SE* = 0.076, *p* < 0.001). For non-theory-based agents, the Crisis Decision-Making dimension differed from the Use of Social Support dimension (estimate = 0.456, *SE* = 0.117, *p* = 0.003), while other comparisons were not statistically significant.

These *Post-hoc* analyses clarify that the interaction effect was primarily driven by higher realism ratings for Theory-Based agents in the Adaptability and Use of Social Support dimensions, and by specific differences among the dimensions within each agent type (theory-based or non-theory-based).

### 4.7 Robustness check: bootstrapped confidence intervals

To assess the robustness of our findings given the modest sample size, we conducted a sensitivity analysis using bootstrapped 95% confidence intervals (10,000 iterations) for the mean differences between theory-based and non-theory-based vignettes across each dimension (see Table 12). The bootstrapped confidence intervals confirm that the observed differences are robust, with all confidence intervals excluding zero for the Crisis Decision-Making [0.12, 0.63], Adaptability [0.35, 0.86], Plausibility of Actions [0.10, 0.67], and Use of Social Support [0.71, 1.23] dimensions. These results provide additional evidence that the effect of theory-based agent design is reliable across dimensions, even with the constraints of our sample size.

**Table 12:** Bootstrapped 95% confidence intervals for theory/non-theory mean differences.

Dimension	Mean difference (theory – non-theory)	Bootstrapped 95% CI
Crisis Decision-Making	0.38	[0.12, 0.63]
Adaptability	0.60	[0.35, 0.86]
Plausibility of Actions	0.38	[0.10, 0.67]
Use of Social Support	0.97	[0.71, 1.23]

Bootstrapping with 10,000 iterations.

## 5 Discussion

### 5.1 Interpretation of findings

The results of the current study provide empirical support for the hypothesis that the behavior of autonomous AI agents grounded in behavioral theory is perceived as more realistic by participants compared to the behavior of agents that lack the same theoretical integration. This finding addresses a key gap in the literature around the validation of digital twin agents, moving beyond technical accuracy to behavior fidelity as perceived by the participants.

Across all four dimensions of realism (crisis decision making, adaptability, plausibility of actions, and use of social support) theory-based agents consistently received higher realism scores. The difference in mean realism ratings between theory-based and non-theory-based agents was statistically significant, with a medium effect size (*Cohen’s d* = 0.54), indicating that the integration of behavioral theory constructs meaningfully enhances the perceived realism of agent behavior in food shortage situations. The robustness of this effect was confirmed through bootstrapped confidence intervals (see Table 12), which demonstrated that theory-based agents were rated higher across all four dimensions even when accounting for sampling variability.

The repeated measures ANOVA further expanded on these findings, revealing significant main effects for both agent type (theory-based vs. non-theory-based) and realism dimension, as well as a significant interaction effect between agent type and dimension. This finding suggests that not only do theory-based agents outperform non-theory-based agents in overall realism, but the magnitude of this effect varies across the different dimensions of realism. Notably, the highest dimension realism score was for the use of social support, reflecting the importance of modeling nuanced social and adaptive behaviors during crisis scenarios and including data that reflects the norms and behavior of relevant social networks. These findings align with prior research indicating that agents are capable of reflecting complex social and psychological processes and that human raters find their behavior more believable and effective in simulated environments (Park et al., 2023).

The findings of the current study demonstrate the utility of FRAME as both a design and assessment tool. By explicitly embedding constructs from HBM, TPB, and SCT into agent logic, and aligning rater evaluation criteria with these constructs, this study represents a replicable method for enhancing and measuring behavioral realism in autonomous agent simulations. This approach addresses a critical gap in the literature, moving beyond rule-based heuristics toward theory-informed agent design that better captures the complexity of human decision-making and behavior under crisis conditions.

### 5.2 Implications of findings

These results have implications for the design, validation, and application of autonomous AI agents in digital twin environments related to food shortages and crisis situations. First, the finding that theory-based agents are consistently rated as more realistic by participants underscores the importance of embedding behavioral theory constructs into agent logic. This approach moves beyond technical or rules-based accuracy and demonstrates that realistic behavior is critical for producing simulations that are valid and can be trusted by researchers. This suggests that integrating established behavioral theories into the agentic AI design can enhance the effectiveness of decision support tools in high-stakes environments.

The implications of these findings also extend to the broader adoptions of simulation models in public health, emergency management, and social policy. As stakeholders increasingly utilize digital twin technology to inform decision-making, the realism of agent behavior is essential for providing accurate simulation outcomes. Transparent, theory-driven agent design can help to ensure that simulation results have utility for decision-makers.

Policymakers weighing digital twins for crisis planning face a fundamental question of trust in simulations. Our findings suggest that grounding agents in behavioral theory establishes not just technical accuracy, but behavioral validity. When agents make decisions reflecting how real people weigh risks and seek support, their actions become interpretable to the experts who must act on simulation results.

This matters most when stakes are high. Emergency managers allocating medical supplies or public health officials targeting interventions need agents that make decisions based on explicit constructs (perceived severity, self-efficacy, social norms) they can recognize and critique. Unlike machine learning models, theory-based logic provides clear rationale that stakeholders can cross-examine directly.

Beyond individual adoption, FRAME offers policy contexts concrete standards. Aligning agent behavior with validated theories and structured evaluation criteria gives oversight bodies benchmarks for assessing digital twin reliability. This standardization could accelerate adoption of AI-driven decision support in public health and emergency management.

### 5.3 Data and code availability

The raw data for realism scores and Python-based simulation code used in this study cannot be included as supplementary materials within this manuscript. However, these materials may be made available to qualified researchers upon request subject to institutional review and approval.

### 5.4 Limitations

There are several limitations to this study. First, participants assessed agent realism through vignettes that summarize an agent’s logged activities throughout the 14-day infant formula shortage. The agent logs are very detailed and lengthy to account for activities during the two-week period. Time constraints necessitated that we use vignettes, which would allow the participants to assess the agents within a limited timeframe. When agents make decisions reflecting how real people weigh risks and seek support, their actions become interpretable to the experts who must act on simulation results. When compared to the agent logs, the vignettes are summaries that do not present each detailed step of how the agents behaved dynamically. Second, since we were focused on evaluating the realism of agents, we did not assess whether theory-based agents would lead to positive behavioral outcomes. While behavioral theory was used to inform agent decision-making, the goal was to assess realism, not desirability. We recognize that more accurate or realistic agent models can be leveraged to drive positive behavioral outcomes, but this depends largely on the policies, constraints, and environmental conditions embedded in the simulation, rather than agent logic alone. Future studies should explore how agent-environment interactions can be designed to support or encourage optimal behaviors in response to crisis conditions. Third, our use case was narrow and only focused on infant formula shortage. Other public health crisis or emergency scenarios may offer different results.

Another limitation is in the lower inter-rater reliability within theory-based vignettes compared to non-theory-based vignettes for the Evaluation Scale, which indicates that more work needs to be done to validate the FRAME tool for differentiating realism within theory-based vignettes. However, the average realism ratings across all vignettes and dimensions of realism (which was used to compare theory-based to non-theory-based vignettes for perceived realism) was high, which indicates the aggregated ratings across raters provides a robust measure for group-level comparisons. This approach is also in line with established practices in social and behavioral science, where mean ratings are often used to mitigate individual rater bias and enhance the reliability of subjective assessments (Bogdanovych et al., 2016; Park et al., 2023).

The current study included 34 participants, which limits statistical power, particularly for detecting smaller effects or interactions. Although the repeated-measures design increases sensitivity by reducing error variance through within-subjects comparisons, and the bootstrapped confidence intervals (see Table 12) confirmed the robustness of our main findings, the small sample size may still lead to underestimation of true effects or the overestimation of effect sizes. A formal *a priori* power analysis was conducted based on expected medium effect sizes (e.g., Cohen’s *d* = 0.5), but it did not fully account for the within-subject correlations across dimensions or the interaction effects observed. Future studies should employ larger samples to improve precision of effect estimates and enable more complex modeling of individual differences in realism perception.

Additionally, our findings may have limited generalizability beyond the specific context studied. This research focused on U. S.-based participants responding to vignettes about an infant formula shortage, which is a high-stress crisis scenario involving vulnerable populations (infants). The extent to which these findings would replicate in non-U.S. populations or less urgent decision-making contexts remains uncertain. Cultural norms around infant feeding practices, social support structures, trust in institutions, and resource availability vary considerably across contexts and may influence both actual caregiver behavior and rater perceptions of what constitutes “realistic” agent behavior. Future work should evaluate behavioral theory-based agents in diverse geographic and socio-cultural settings, across different types of crises (e.g., natural disasters, infectious disease outbreaks, economic disruptions), and under varying levels of urgency to test both the validity of these behavioral theories and the robustness of theory-grounded agent design.

A final limitation concerns potential AI model bias. Even with the grounding of the autonomous agents with established behavioral theories and using structure human rating for the evaluation, this does not eliminate the possibility of bias, which can result from issues such as vignette framing, theory selection, and sociocultural assumptions in the simulation environment that does not guarantee equitable representation across populations or normative correctness. The study included multiple behavioral theories, construct mapping, and standardized evaluation criteria to mitigate some of these risks. However, future research should include bias audits as part of digital twin workflows (Gao et al., 2024; Ranjan et al., 2025).

## 6 Conclusion

Our study demonstrates that when autonomous AI agents are designed with behavioral theory, they are perceived as more realistic in how they make decisions and interact with others. In the simulated infant formula shortage scenario, theory-based agents consistently aligned more closely with human expectations than non-theory-based agents. This finding emphasizes the importance of embedding behavioral science theory into the next generation of digital twins.

Equally important, we have shown that it is possible to test and refine the realism of these agents in a systematic way. By introducing a method for evaluating behavioral fidelity, we take a first step toward building simulation environments that people can trust to reflect real-world decision-making under crisis conditions. Such credibility is critical if digital twins are to be used in high stakes planning and policy.

Looking ahead, this work opens a path for expanding both the scope and depth of AI agents that are informed by behavioral theory. Future studies can extend the approach to different populations, diverse crises, and more complex social dynamics. As policymakers and researchers increasingly rely on AI-driven simulation for strategic planning, this research provides both an approach and a scientific foundation for ensuring that autonomous agents remain grounded in human reality.

### 6.1 Future work

There are several considerations for future research that could build on this study’s findings. One consideration for future research is to conduct a longitudinal evaluation running longer simulations and direct behavioral assessments. Park et al. (2023) recommend studying agents for extended periods of time to help set benchmarks for how believable they are. Another consideration is empirical validation comparing simulated caregiver agent behavior to data from real caregivers during the infant formula shortage of 2022. Leveraging the data from human caregivers more fully in the design of the caregiver agents can enhance their realism with higher accuracy. A third consideration is to build autonomous population agents employing a weighted decision-making framework that uses 14 evidence-based behavioral factors drawn from recent behavioral science research (Cane et al., 2012; Zhou et al., 2024). In that research, the decision weights were adjusted according to emotional states like fear, urgency, and hope, reflecting research that shows emotions significantly affect decision-making when dealing with health risks or crises (Ferrer et al., 2017; Harper et al., 2021).

An important direction for future research involves validating FRAME across the diverse application domains discussed above. Extending this methodology to defense scenarios, healthcare systems, and urban planning contexts would test its generalizability and identify domain-specific adaptations needed for optimal performance. For example, military planners use agent-based models to predict civilian responses during conflicts or disasters. Theory-based agents would provide more realistic simulations of displacement and resource-seeking behaviors, letting analysts assess whether patterns match documented crises. In healthcare, digital twins could simulate patient decisions around treatment adherence and preventive care. Urban planners could use theory-based agents to simulate responses to transit changes or new services, with TPB informing transportation choices and SCT modeling neighborhood influences.

Finally, the use of human raters and the FRAME tool provide a replicable and structured method for evaluating agent realism. FRAME aligns behavioral theory with agent behavior constructs and rater evaluation dimensions. It sets a foundation for advancing the design and evaluation of AI agents and AI-driven digital twins. This addresses a gap in the field, where validation efforts have often focused on internal consistency, technical performance, or rules-based evaluation rather than the external perceived realism of agent behavior by human raters (Bogdanovych et al., 2016). By aligning agent design and evaluation criteria with behavioral theory, this framework offers a pathway for standardizing the validation process across domains and ensuring that autonomous agents are technically robust, ecologically valid, and perceived as realistic.

## Data Availability

The data supporting the conclusions of this study are not publicly available due to internal MITRE restrictions. De‑identified data and supporting materials may be made available from the corresponding author upon reasonable request and with MITRE approval.
