# Correction: The realism of behavioral theory-based vs. non-theory-based AI agents during a simulated infant formula shortage

**DOI:** 10.3389/frai.2026.1812447

**Published:** 2026-04-22

**Authors:** Linda Desens, Brandon Walling, Rhys O'Neill, Vanessa Howard, Mary Giammarino, Denise Scannell, Anya Kemble, Taylor Wilkerson, Nyalok Nhial, Sara Beth Elson, Maureen Leahy, Scott Rosen

**Affiliations:** The MITRE Corporation, McLean, VA, United States

**Keywords:** agentic AI, AI agent, AI agent validation, autonomous agents, behavioral theory, digital twins

Author Maureen Leahy (ML) was omitted as an author in the published article. The correct author list reads:

Linda Desens, The MITRE Corporation

Brandon Walling, The MITRE Corporation

Rhys O'Neill, The MITRE Corporation

Vanessa Howard, The MITRE Corporation

Mary Giammarino, The MITRE Corporation

Denise Scannell, The MITRE Corporation

Anya Kemble, The MITRE Corporation

Taylor Wilkerson, The MITRE Corporation

Nyalok Nhial, The MITRE Corporation

Sara Beth Elson, The MITRE Corporation

Maureen Leahy, The MITRE Corporation

Scott Rosen, The MITRE Corporation

The **Author Contributions** Statement has been corrected to read:

LD: Formal analysis, Data curation, Methodology, Writing – review & editing, Writing – original draft, Investigation, Conceptualization, Funding acquisition, Project administration, Resources, Supervision, Validation. BW: Formal analysis, Writing – original draft, Methodology, Data curation, Writing – review & editing, Investigation. RO'N: Writing – review & editing, Conceptualization, Methodology, Writing – original draft, Visualization, Data curation. VH: Project administration, Writing – review & editing, Data curation, Writing – original draft, Methodology. MG: Investigation, Writing – review & editing, Project administration, Writing – original draft. DS: Writing – original draft, Funding acquisition, Conceptualization, Writing – review & editing. TW: Writing – original draft, Data curation. AK: Data Curation, Writing – original draft. NN: Writing – original draft. SE: Methodology, Writing – original draft. ML: Writing-original draft, Writing – review & editing. SR: Writing – original draft, Project administration, Supervision, Writing – review & editing.

The **Conflict of interest** Statement has been corrected to read:

LD, R'ON, BW, VH, MG, DS, SE, TW, AK, NN, ML, and SR were employed by The MITRE Corporation. The MITRE Corporation's Independent Research and Development program provided internal support for the study design, data collection, analysis, and manuscript preparation.

The **Citation** Statement has been corrected to read:

Citation: Desens L, Walling B, O'Neill R, Howard V, Giammarino M, Scannell D, Kemble A, Wilkerson T, Nhial N, Elson SB, Leahy M and Rosen S.

The **Copyright** Statement has been corrected to read:

Copyright: Desens, Walling, O'Neill, Howard, Giammarino, Scannell, Kemble, Wilkerson, Nhial, Elson, Leahy and Rosen.

A correction has been made to the **Introduction** section, paragraphs 4 and 5. The paragraphs previously read:

This study highlights the importance of incorporating behavioral theory in the design of AI agents. AI agent modeling and simulation should advance from a rule-based decision tree to agent designs that are grounded in empirically validated behavioral theory. To achieve greater realism, agents should be designed with socially adaptive mechanisms that enable them to adjust their strategies based on evolving human norms, values and behaviors to reflect realistic human behavior (Rahwan et al., 2019).

and internal processes such as cooperation that reflect human behavior (Diau, 2025). Agents should include reflective reasoning, goal representations, memory, and behavioral theoretical grounding to maintain consistency and behavioral coherency over time (Park et al., 2023). Capturing these nuances in a simulated environment is essential to ensure that autonomous AI agents realistically reflect the decision-making patterns of the populations they represent. In this study, incorporating well-established behavioral theories into agent design ensures that simulated actions are grounded in how people react during a crisis.

The paragraph has been corrected to read:

This study highlights the importance of incorporating behavioral theory in the design of AI agents. AI agent modeling and simulation should advance from a rule-based decision tree to agent designs that are grounded in empirically validated behavioral theory. To achieve greater realism, agents should be designed with socially adaptive mechanisms that enable them to adjust their strategies based on evolving human norms, values and behaviors to reflect realistic human behavior (Rahwan et al., 2019) and internal processes such as cooperation that reflect human behavior (Diau, 2025). Agents should include reflective reasoning, goal representations, memory, and behavioral theoretical grounding to maintain consistency and behavioral coherency over time (Park et al., 2023). Capturing these nuances in a simulated environment is essential to ensure that autonomous AI agents realistically reflect the decision-making patterns of the populations they represent. In this study, incorporating well-established behavioral theories into agent design ensures that simulated actions are grounded in how people react during a crisis.

The original version of this article has been updated.

