# Supplementary material for: The realism of behavioral theory-based vs. non-theory-based AI agents during a simulated infant formula shortage
Source: Front Artif Intell. 2026 Feb 9;9:1719703. doi: 10.3389/frai.2026.1719703 (PMC12926465; doi:10.3389/frai.2026.1719703)
Supplement: Supplementary file 1 [file Data_Sheet_1.docx]

Appendix: Vignette Examples

**Example 1:**

Sociodemographic Profile

- Location: South Dallas County, Texas
- Household Type: Single mother with two children (one 10-month-old, one 5-year-old)
- Income Level: Low Income
- WIC Eligible: Yes
- Transportation: No personal vehicle, relies on rideshares and occasional family support
- Support Network: Moderate (sister nearby, participates in local parenting group)
- Financial Situation: Low-income, depends on WIC and SNAP benefits

Vignette
Angela lives in South Dallas County with her 10-month-old baby and 5-year-old son. When the infant formula shortage begins, she feels overwhelmed but starts by assessing her situation carefully. On day one, she has about six days of formula. She uses WIC benefits to add enough for an extra week and reaches out to her sister for backup support if needed. During the first week, Angela combines multiple strategies to maintain her supply. She times store visits after checking online restock notifications, coordinates with her sister to share resources, and joins a local parenting group that posts updates about which stores still have formula available. By day six, she has built up a 15-day reserve, easing her initial anxiety. By the middle of the infant formula shortage, Angela begins conserving formula while still making proactive store trips. When stock is unavailable locally, she travels farther using rideshares and coordinates pickups with her sister. She also contacts her WIC office to confirm alternate suppliers and uses community tips to find less-known stores with formula in stock. By the end of the two weeks, Angela has managed to secure enough formula to last about three weeks. While she still worries about future shortages, her stress has leveled off thanks to deliberate planning and social coordination. By day fourteen, her careful mix of WIC benefits, community updates, and flexible decision-making has kept her baby healthy and fed.

**Example 2:**

Sociodemographic Profile

- Location: South Dallas County, Texas
- Household Type: Single mother with two children (one 10-month-old, one 5-year-old)
- Income Level: Low Income
- WIC Eligible: Yes
- Transportation: No personal vehicle, relies on rideshares and occasional family support
- Support Network: Moderate (sister nearby, participates in local parenting group)
- Financial Situation: Low-income, depends on WIC and SNAP benefits

Vignette
Angela lives in South Dallas County with her 10-month-old baby and 5-year-old son. During the infant formula shortage, she bases her decisions entirely on how much formula she has left. On the first day, she has plenty on hand, so she decides to wait before buying more. By day four, Angela’s formula runs low, so she goes to the store and buys just enough to get her supply back up. During the first week, the same pattern keeps happening: when her formula runs low, she makes a store trip; when she has enough, she waits. By the middle of the infant formula shortage, Angela has made several short trips to nearby stores but only buys enough to stay slightly above her comfort level. There’s no bigger plan involved. If a store is out of formula, she leaves without buying anything and waits until her supply drops again before trying another store. By day fourteen, Angela still has a little formula left, but just barely above the amount where she starts worrying. Whether she has enough depends completely on what’s available when she shops. She hasn’t run out, but she also hasn’t built up any extra for the days ahead.
